# Non-invasive assessment of Pulse Wave Transit Time (PWTT) is a poor predictor for intraoperative fluid responsiveness: a prospective observational trial (best-PWTT study)

**DOI:** 10.1186/s12871-023-02016-0

**Published:** 2023-02-27

**Authors:** Kimiko Fukui, Johannes M. Wirkus, Erik K. Hartmann, Irene Schmidtmann, Gunther J. Pestel, Eva-Verena Griemert

**Affiliations:** 1 Department of Anesthesiology, University Medical Center of the Johannes Gutenberg-University, Langenbeckstraße 1, 55131 Mainz, Germany; 2 Institute for Medical Biostatistics, Epidemiology and Informatics Medical (IMBEI), University Medical Center of the Johannes Gutenberg-University, Mainz, Germany

**Keywords:** Pulse wave transit time, Fluid responsiveness, Hemodynamic monitoring, Fluid resuscitation, Pulse pressure variation

## Abstract

**Background:**

Aim of this study is to test the predictive value of Pulse Wave Transit Time (PWTT) for fluid responsiveness in comparison to the established fluid responsiveness parameters pulse pressure (ΔPP) and corrected flow time (FTc) during major abdominal surgery.

**Methods:**

Forty patients undergoing major abdominal surgery were enrolled with continuous monitoring of PWTT (LifeScope® Modell J BSM-9101 Nihon Kohden Europe GmbH, Rosbach, Germany) and stroke volume (Esophageal Doppler Monitoring CardioQ-ODM®, Deltex Medical Ltd, Chichester, UK). In case of hypovolemia (difference in pulse pressure [∆PP] ≥ 9%, corrected flow time [FTc] ≤ 350 ms) a fluid bolus of 7 ml/kg ideal body weight was administered. Receiver operating characteristics (ROC) curves and corresponding areas under the curve (AUCs) were used to compare different methods of determining PWTT. A Wilcoxon test was used to discriminate fluid responders (increase in stroke volume of ≥ 10%) from non-responders. The predictive value of PWTT for fluid responsiveness was compared by testing for differences between ROC curves of PWTT, ΔPP and FTc using the methods by DeLong.

**Results:**

AUCs (area under the ROC-curve) to predict fluid responsiveness for PWTT-parameters were 0.61 (raw c finger Q), 0.61 (raw c finger R), 0.57 (raw c ear Q), 0.53 (raw c ear R), 0.54 (raw non-c finger Q), 0.52 (raw non-c finger R), 0.50 (raw non-c ear Q), 0.55 (raw non-c ear R), 0.63 (∆ c finger Q), 0.61 (∆ c finger R), 0.64 (∆ c ear Q), 0.66 (∆ c ear R), 0.59 (∆ non-c finger Q), 0.57 (∆ non-c finger R), 0.57 (∆ non-c ear Q), 0.61 (∆ non-c ear R) [raw measurements vs. ∆ = respiratory variation; c = corrected measurements according to Bazett’s formula vs. non-c = uncorrected measurements; Q vs. R = start of PWTT-measurements with Q- or R-wave in ECG; finger vs. ear = pulse oximetry probe location]. Hence, the highest AUC to predict fluid responsiveness by PWTT was achieved by calculating its respiratory variation (∆PWTT), with a pulse oximeter attached to the earlobe, using the R-wave in ECG, and correction by Bazett’s formula (AUC best-PWTT 0.66, 95% CI 0.54–0.79). ∆PWTT was sufficient to discriminate fluid responders from non-responders (*p* = 0.029). No difference in predicting fluid responsiveness was found between best-PWTT and ∆PP (AUC 0.65, 95% CI 0.51–0.79; *p* = 0.88), or best-PWTT and FTc (AUC 0.62, 95% CI 0.49–0.75; *p* = 0.68).

**Conclusion:**

ΔPWTT shows poor ability to predict fluid responsiveness intraoperatively. Moreover, established alternatives ΔPP and FTc did not perform better.

**Trial registration:**

Prior to enrolement on clinicaltrials.gov (NC T03280953; date of registration 13/09/2017).

**Supplementary Information:**

The online version contains supplementary material available at 10.1186/s12871-023-02016-0.

## Background

Perioperative fluid therapy has a significant impact on patients’ outcome [1–4]. Unfortunately, fluid administration can be too early, too late, too restrictive or too liberal [5]. Keeping fluid homeostasis within an optimal range, however, improves outcome and has an economic benefit, as perioperative morbidity is associated with increasing costs [6].

Consequently, targeted fluid management needs adequate monitoring. Although different approaches to monitor fluid status have been described [7], assessing fluid responsiveness [8] is considered by most to be the crucial first step. Therefore, a reliable, non-invasive, continuous, and reusable monitoring technology would have outcome-relevant and economic benefit.

Pulse wave transit time (PWTT) is the time period from the R-wave in ECG to the upstroke of the pulse plethysmographic waveform or the arterial pressure curve. Its respiratory variation is a flow-based monitoring parameter and was shown to predict fluid responsiveness in sedated ICU patients [9]. Furthermore, respiratory variation of pre-ejection period (∆PEP), a fraction of PWTT, predicted fluid responsiveness in a similar population [10].

The role of PWTT in guiding intraoperative fluid management has not been established. We therefore conducted a prospective monocentric observational clinical study to first, identify which way of assessing PWTT would achieve the highest AUC for prediction of fluid responsiveness (best-PWTT). Second, we compared best-PWTT to difference in pulse pressure (∆PP) and corrected flow time (FTc), both validated fluid responsiveness monitoring parameters.

## Methods

With approval from the State ethics committee of Rhineland-Palatinate, Germany (authorization number 837.004.16) and after obtaining written informed consent, we enrolled patients at the Department of Anesthesiology of the University Medical Center Mainz, Germany. We conducted the study in accordance with the declaration of Helsinki 1996 and ICH guideline E6 (Good Clinical Practice) and registered the protocol prior to enrolling the first patient on clinicaltrials.gov (NC-T03280953; date of registration 13/09/2017).

This manuscript adheres to the applicable TRIPOD guidelines [11].

### Patients

We enrolled non-pregnant patients with an ASA-PS 1–3 and a BMI < 35 kg/m^2^, scheduled for major abdominal surgery with an expected higher intraoperative fluid turnover. Exclusion criteria were symptomatic vascular disease, cardiac rhythm other than sinus, symptomatic cardiac valve disease, restrictive lung disease, and sepsis. Also, patients suffering from esophageal disease of any kind were excluded.

### Patient management and fluid therapy

We placed all patients on standard non-invasive monitors. Anesthesia was induced according to the standard operating procedures [SOP] of our hospital using 1.5–2.5 mg/kg body weight (BW) Propofol, 0.2–0.5 µg/kg BW Sufentanil and 0.5–0.6 mg/kg ideal body weight (IBW) Atracurium or 0.6 mg/kg IBW Rocuronium at the discretion of the attending anesthesiologist. Maintenance of anesthesia was accomplished using Sevoflurane adjusted to age-corrected mean alveolar concentration. Analgesia was adapted to meet patient individual demands using additional Sufentanil boluses of 5–10 µg. A non-opioid analgetic was administered 30 min before the end of the procedure for early post-operative analgesia.

An arterial cannula was placed in the radial artery for continuous, invasive blood pressure monitoring (Philips IntelliVue MX700, Philips Medizin Systeme GmbH, Boeblingen, Germany). Invasive blood pressure monitoring was calibrated by placing the pressure transducer at the level of the right atrium and venting the transducer to atmospheric pressure for reference. ∆PP was calculated as

$$\mathrm{\Delta PP }[\mathrm{\%}] (({\mathrm{PP}}_{\mathrm{max}} - {\mathrm{PP}}_{\mathrm{min}})/ ([{\mathrm{PP}}_{\mathrm{max}} + {\mathrm{PP}}_{\mathrm{min}}]/2)) \times 100$$

where PP_max_ and PP_min_ are the maximum and minimum pulse pressure during one respiratory cycle. ΔPP measurements were calculated from beat-to-beat arterial pressure values and reported as the average value of the last 32 s.

Stroke volume was measured by esophageal doppler monitoring (CardioQ-ODM®, Deltex Medical Ltd, Chichester, UK). The tip of the doppler probe was placed in the pars thoracica of esophagus at the level of the descending aorta. Stroke volume was calculated by the product of stroke distance (area of the doppler derived velocity–time waveform) and aortic cross-sectional area [12].

Patients were mechanically ventilated with a tidal volume ≥ 8 ml/kg IBW using pressure-controlled ventilation. Positive end-exspiratory pressure was kept between 5–8 mbar, respiratory rate was set to 12–16 min^−1^ and fraction of inspired oxygen (F_i_O_2_) was set to keep oxygen saturation above 95% according to departmental standard operating procedures. All settings and target values were continuously adapted to patient characteristics and clinical situation using repetitive blood gas analyses.

In case of possible hypovolemia a fluid bolus of 7 ml/kg IBW was administered at the discretion of the attending anesthesiologist.

Trigger for fluid bolusing were:

- Difference in pulse pressure [∆PP] ≥ 9% [13, 14] and/or
- corrected flow time [FTc] ≤ 350 ms [15]

An increase in stroke volume ≥ 10% following the fluid bolus was considered ‘fluid responsive’.

### Measurements

Pulse wave transit time (PWTT) was assessed with a 6-poled-ECG and pulse oximeter probes placed at the fingertip and the earlobe (LifeScope® model J BSM-9101 Nihon Kohden Europe GmbH, Rosbach, Germany).

For the purposes of this study, we assessed PWTT based on 16 different combinations of the following variables:

1. Beginning of PWTT was either defined by the R- wave (R) or the Q-wave (Q) in ECG.
2. End of PWTT was assessed by the upstroke of the plethysmography wave of a pulse oximeter attached either to the fingertip (finger) or the earlobe (ear).
3. Measurements were corrected (c) according to Bazett’s formula [16] or left uncorrected (non-c).
4. Respiratory variation of PWTT (∆PWTT) was calculated or PWTT was analysed as measured (raw).

Simultaneous measurements of all 16 PWTT parameters in addition to ∆PP, FTc, stroke volume (SV), cardiac output (CO), mean arterial pressure (MAP) and heart frequency (HF) were recorded immediately before and 1 min after completion of each fluid bolus.

Pulse oximetry waveforms and ECG waveforms were recorded with sampling periods of 8 ms and 4 ms, respectively. The rising point of the plethysmography waveform representing pulse wave arrival was defined as the point where the differentiated signal reaches 30% of the peak value of the derivative [17]. The respiratory variation of PWTT (∆PWTT) was calculated by the following formula:

$$\mathrm{\Delta PWTT }= ({\mathrm{PWTT}}_{\mathrm{max}} - {\mathrm{PWTT}}_{\mathrm{min}})/{\mathrm{PWTT}}_{\mathrm{mean}}$$

where PWTT_max_ and PWTT_min_ are the maximum and minimum values of PWTT within 20 s and PWTT_mean_ is the average value of PWTT within the last 20 s.

### Statistical analysis

#### Sample size consideration

This study was an exploratory study aiming at obtaining tentative signals which PWTT parameters might be useful to predict fluid response. Sample size was primarily guided by feasibility. The number of patients to be included during the course of one year was expected to be about 40, providing about 80 measurements with subsequent bolus. Assuming that fluid response would occur in roughly half of the patients, we would expect approximately 40 responses and 40 non-responses. To test whether the area under the curve (AUC) of an obtained receiver operating characteristics (ROC) curve is higher than 0.5 a one-sided Wilcoxon Mann Whitney test can be applied. With 40 responses and 40 non-responses it is possible to reject the null hypothesis at the 5% significance level with 80% power if the true AUC is at least 0.662. Thus, the planned sample size was sufficient to identify possibly useful PWTT parameters.

#### Missing values

We performed a complete case analysis including only datasets without missing data on the variables needed for analysis of the primary endpoint (prediction of fluid responsiveness). However, multiple imputations were calculated to prevent bias by analysing complete cases only [18].

The proportion of missing data ranged from 5.6% (raw non-c finger R) to 26% (∆ non-c ear R). There were no obvious systematic patterns in missing data. Estimates obtained using multiple imputation deviated by only 3.3% on average. When restricting the analysis to complete cases only, the same PWTT-parameter achieved the highest AUC for prediction of fluid responsiveness (best-PWTT) as deviation of best-PWTT after multiple imputations from corresponding mean in complete case analysis was 0.18% only.

#### Primary analysis

The ability to predict fluid responsiveness for the 16 different PWTT parameters assessed in this study was described by the area under receiver operating characteristic curve (AUC). Differences between ROC curves were analysed using the methods by DeLong [19]. A parameter was considered to have an excellent diagnostic value when AUC was higher than 0.90, good diagnostic value when AUC was between 0.75 and 0.90, poor diagnostic value when AUC was between 0.50 and 0.75 and no diagnostic value when AUC was lower than 0.50 [20].

Differences in stroke volume change after fluid bolusing between responders and non-responders were assessed using a two-sided Wilcoxon test.

#### Secondary analysis

After identifying which way of assessing PWTT achieved the highest AUC for prediction of fluid responsiveness (best-PWTT), we compared AUC of best-PWTT with AUCs of ∆PP and FTc using the methods by DeLong [19] to test for differences in the ability to predict fluid responsiveness.

#### Sensitivity analysis

Positive predictive value was calculated for all fluid responsiveness parameters investigated, i.e. 16 PWTT-parameters as well as ∆PP and FTc. As both, administering no fluids despite they are needed as well as to do so but at the wrong time can be harmful, only positive predictive values with sensitivity of at least 80% are displayed in this analysis. Furthermore, we calculated the F1-Score thus balancing the importance of sensitivity and positive predictive value. We used precision recall curves to illustrate relation of sensitivity and positive predictive value (Fig. 4 Appendix).

SAS 9.4 (SAS Institute Corp., USA) was used for all statistical analyses. Boxplots were generated using Excel 2016 (Microsoft Corp., USA).

## Results

Eighty patients were scheduled for major abdominal surgery with an expected higher intraoperative fluid turnover. 40 patients did not meet the inclusion criteria. The remaining 40 patients consented and were enrolled in the study. 1 patient was excluded due to not receiving a fluid bolus and 1 patient because of incomplete documentation.

In total, data of 38 patients were analyzed. Patients’ demographics are shown in Table 1, and procedures performed are summarized in Table 2.

**Table 1:** Patients’ demographics

Characteristic	Mean (standard deviation)
age [years]	60.2 (9.0)
m:f [n]	29:9
height [cm]	175.7 (11.3)
weight [kg]	82.3 (16.9)
BMI [kg/m^2^]	26.6 (4.8)
ASA-PS (I/ II/ III/ IV)	0/ 23/ 15/ 0
hypertension [n]	9

(age, gender [male:female], height, weight, BMI, ASA physical status classification and pre-existing arterial hypertension)

**Table 2:** Surgical Diagnoses

Procedure performed	n
Cystectomy	15
Nephrectomy	10
Prostatectomy	6
Segmental colectomy	3
Whipple procedure	2
Pyeloplasty	1
Urethral reimplantation	1

(procedures performed and corresponding count [n])

All procedures were performed in laparotomy. Patients receiving a laparoscopy respectively the use of pneumoperitoneum were not included in the study.

Out of 85 fluid boluses administered to 38 patients, 74 datasets were eligible for complete case analysis. We identified 28 out of 38 patients as fluid responders, fluid responsiveness rate was 67.6% (50 out of 74 fluid boluses given).

Hemodynamic measurements before and after fluid bolus separated for fluid responders and non-responders are shown in Table 3.

**Table 3:** Hemodynamic measurements before and after fluid bolus

parameter	before fluid bolus		after fluid bolus	
	**fluid responder**	**non-responder**	**fluid responder**	**non-responder**
**PWTT raw c finger Q**	290.4	273.1	272.1	270.3
**PWTT raw c finger R**	254.5	242.2	237.4	238.2
**PWTT raw c ear Q**	195.6	187.6	177.7	176.5
**PWTT raw c ear R**	159.1	156.9	142.4	146.2
**PWTT raw non-c finger Q**	268.6	262.8	253.7	253.5
**PWTT raw non-c finger R**	235.4	233.1	221.2	223.5
**PWTT raw non-c ear Q**	180.6	180.7	165.4	172.7
**PWTT raw non-c ear R**	146.6	151.2	132.4	143.2
**PWTT Δ c finger Q**	13.3	8.6	10.8	9.1
**PWTT Δ c finger R**	9.1	5.9	7.5	5.9
**PWTT Δ c ear Q**	13.1	8.0	11.7	7.6
**PWTT Δ c ear R**	9.3	4.5	7.7	4.3
**PWTT Δ non-c finger Q**	7.6	6.1	6.8	6.3
**PWTT Δ non-c finger R**	6.1	4.3	5.1	4.2
**PWTT Δ non-c ear Q**	12.0	8.3	10.2	10.5
**PWTT Δ non-c ear R**	10.9	5.6	7.7	8.2
**∆ PP [%]**	13.3	10.3	7.5	7.0
**FTc [ms]**	313.7	332.0	352.2	347.9
**CO [l/min]**	4.9	5.5	6.3	5.6
**MAP [mmHg]**	69.4	67.6	76.1	75.5
**HF [min**^**−1**^**]**	70.2	66.0	69.0	66.7

Mean of hemodynamic measurements before and after fluid bolus, displayed separately for fluid responders (= increase in stroke volume of ≥ 10%) and non-responders (< 10%). PWTT raw measurements in [ms] vs. ∆ = respiratory variation in [%]; c = corrected measurements according to Bazett’s formula vs. non-c = uncorrected measurements; Q vs. R = start of PWTT-measurements with Q- or R-wave in ECG; finger vs. ear = pulse oximetry probe location; ∆PP = difference in pulse pressure in [%]; *FTc* corrected flow time in [ms], *CO* cardiac output in [litre/min], *MAP* mean arterial pressure in [mmHg], *HF* heart frequency in [min^−1^]

### Primary analysis: comparison of AUC for prediction of fluid responsiveness between PWTT variants

Of all 16 different ways to assess PWTT, a measurement of ∆PWTT with use of the R-wave in ECG, plethysmography at the earlobe, and correction by Bazett’s formula performed best to predict fluid responsiveness (‘best-PWTT’ AUC 0.66, SD 0.06, 95% CI 0.54–0.79), and was able to discriminate fluid responders from non-responders (*p* = 0.029).

**Fig. 1:**
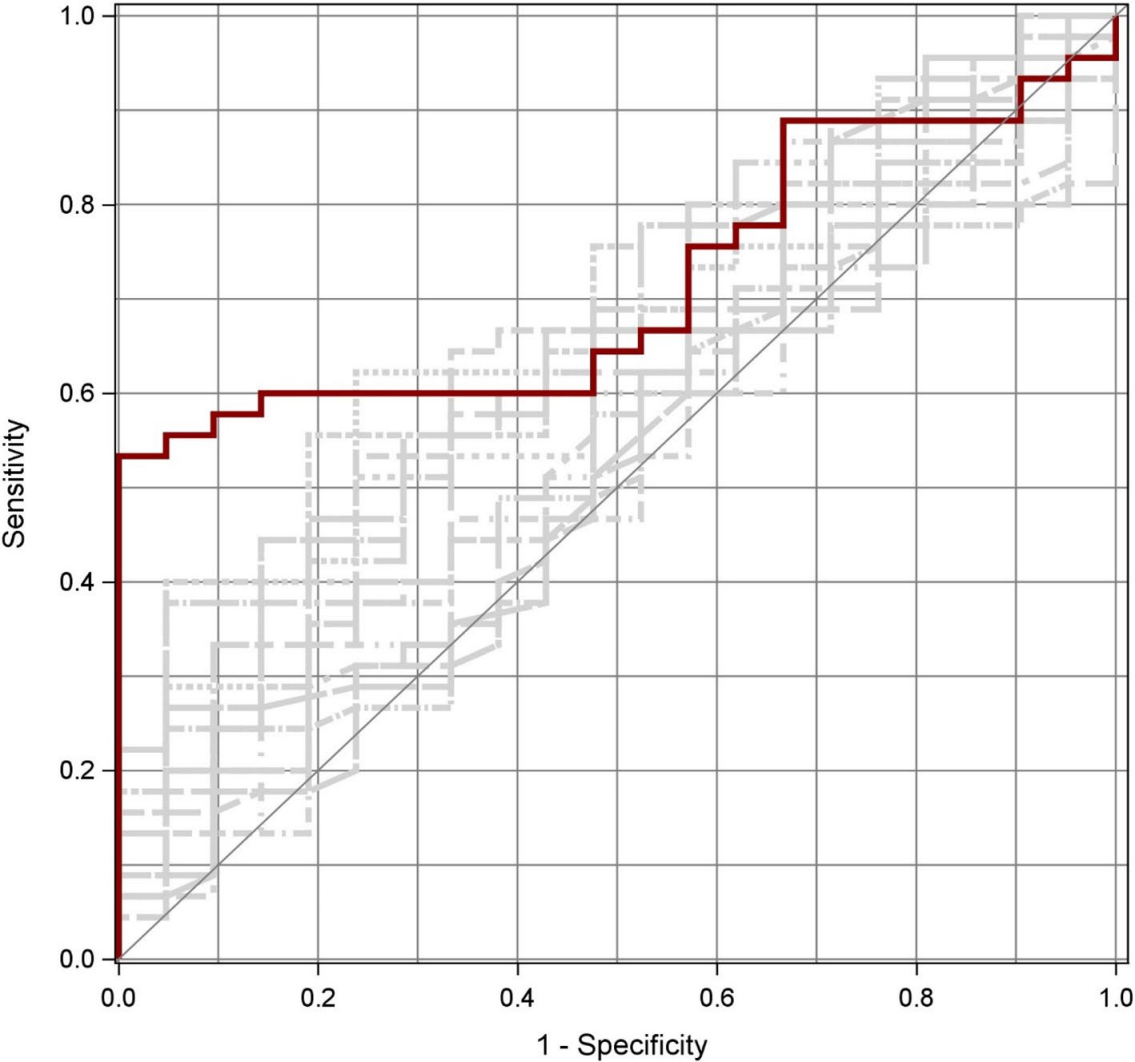
ROC-curve of best-PWTT. Sensitivity and specificity to predict fluid responsiveness (= increase in stroke volume ≥ 10%)

Figure 1 shows the AUC of best-PWTT as described above in bold red in the front. For comparison, AUCs of all other 15 PWTT variants (s. 2.3.) performed worse and are therefore shown hatched in the background.

ROC analyses with standard deviation and confidence interval of all 16 PWTT-variants investigated are shown in numbers in Table 4. P-values of the Wilcoxon test for differences between PWTT-measurements before fluid responsiveness and PWTT-measurements before non-responsiveness are shown in Table 4.

**Table 4:** ROC models and Wilcoxon-test of all 16 PWTT-parameters

ROC model	AUC	SD	95% CI	*P*-value
PWTT raw c finger Q	0.61	0.07	0.48—0.75	0.131
PWTT raw c finger R	0.61	0.07	0.48—0.74	0.095
PWTT raw c ear Q	0.57	0.07	0.43—0.70	0.439
PWTT raw c ear R	0.53	0.07	0.39—0.67	0.598
PWTT raw non-c finger Q	0.54	0.07	0.39—0.68	0.790
PWTT raw non-c finger R	0.52	0.07	0.37—0.66	0.925
PWTT raw non-c ear Q	0.50	0.07	0.36—0.65	0.727
PWTT raw non-c ear R	0.55	0.07	0.41—0.69	0.490
∆PWTT c finger Q	0.63	0.07	0.50—0.76	0.091
∆PWTT c finger R	0.61	0.07	0.48—0.74	0.174
∆PWTT c ear Q	0.64	0.06	0.51—0.77	0.091
*∆PWTT c ear R (best-PWTT)*	*0.66*	*0.06*	*0.54—0.79*	*0.029*
∆PWTT non-c finger Q	0.59	0.07	0.44—0.74	0.237
∆PWTT non-c finger R	0.57	0.07	0.43—0.71	0.394
∆PWTT non-c ear Q	0.57	0.07	0.42—0.71	0.623
∆PWTT non-c ear R	0.61	0.07	0.47—0.75	0.280

raw measurements in [ms] vs. ∆ = respiratory variation in [%]; c = corrected measurements according to Bazett’s formula vs. non-c = uncorrected measurements; Q vs. R = start of PWTT-measurements with Q- or R-wave in ECG; finger vs. ear = pulse oximetry probe location. *P*-value of Wilcoxon-test to discriminate fluid responders from non-responders (= increase in stroke volume ≥ 10%). *n* = 74

Positive Predictive value and F1-Score of all 16 PWTT parameters are displayed in Table 5. Depending on different sensitivity (each of at least 80%), mean, minimum and maximum positive predictive value of all parameters have been calculated.

**Table 5:** Mean, Minimum and Maximum Positive Predictive Value with Sensitivity > 80% and F1-Score of all 16 PWTT-parameters

Parameter	Mean	Min	Max	F1-score
PWTT raw c finger Q	0.69	0.66	0.73	0.806
PWTT raw c finger R	0.67	0.66	0.68	0.806
PWTT raw c ear Q	0.67	0.66	0.68	0.806
PWTT raw c ear R	0.66	0.64	0.68	0.806
PWTT raw non-c finger Q	0.68	0.66	0.70	0.806
PWTT raw non-c finger R	0.69	0.67	0.71	0.806
PWTT raw non-c ear Q	0.68	0.67	0.69	0.806
PWTT raw non-c ear R	0.67	0.66	0.69	0.810
∆PWTT c finger Q	0.69	0.68	0.71	0.817
∆PWTT c finger R	0.68	0.67	0.70	0.806
∆PWTT c ear Q	0.68	0.67	0.70	0.820
*∆PWTT c ear R (best-PWTT)*	*0.69*	*0.67*	*0.73*	*0.806*
∆PWTT non-c finger Q	0.71	0.67	0.75	0.814
∆PWTT non-c finger R	0.68	0.66	0.69	0.806
∆PWTT non-c ear Q	0.70	0.68	0.72	0.821
∆PWTT non-c ear R	0.70	0.66	0.74	0.813

raw measurements in [ms] vs. ∆ = respiratory variation in [%]; c = corrected measurements according to Bazett’s formula vs. non-c = uncorrected measurements; Q vs. R = start of PWTT-measurements with Q- or R-wave in ECG; finger vs. ear = pulse oximetry probe location. *n* = 74

F1-score is the mean of positive predictive value and sensitivity, showing values closer to 1 if both, sensitivity and positive predictive value reach their maximum.

**Fig. 2:**
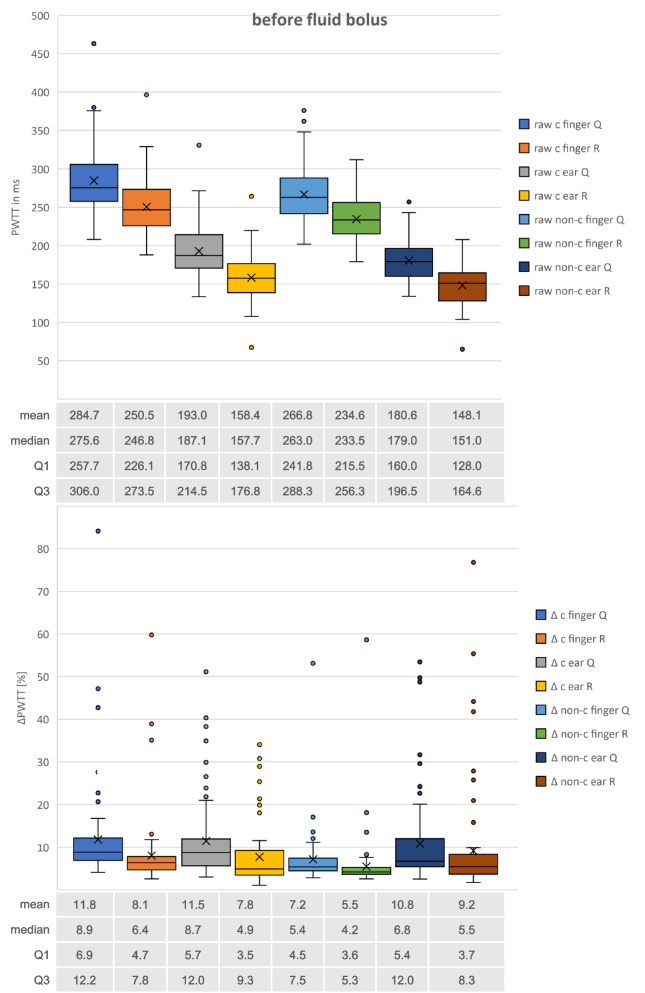
Boxplots of all PWTT measurements before fluid bolus. Raw measurements in [ms] vs. ∆ = respiratory variation in [%]; c = corrected measurements according to Bazett’s formula vs. non-c = uncorrected measurements; Q vs. R = start of PWTT-measurements with Q- or R-wave in ECG; finger vs. ear = pulse oximetry probe location. Mean, median, Q1 = 1st, Q2 = 3rd quartile of PWTT-measurements

**Fig. 3:**
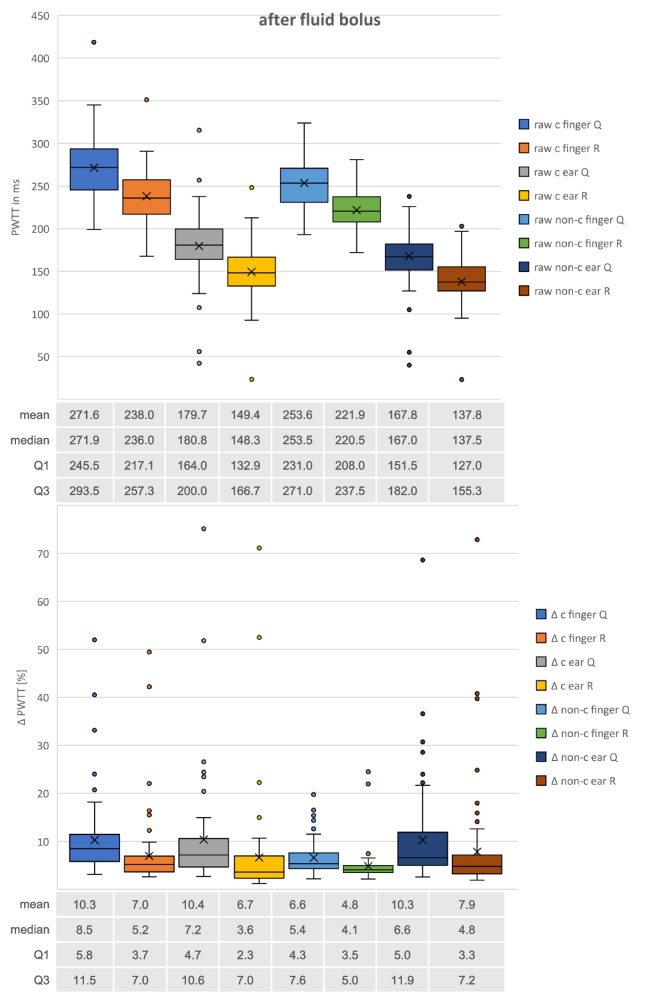
Boxplots of all PWTT measurements after fluid bolus. Raw measurements in [ms] vs. ∆ = respiratory variation in [%]; c = corrected measurements according to Bazett’s formula vs. non-c = uncorrected measurements; Q vs. R = start of PWTT-measurements with Q- or R-wave in ECG; finger vs. ear = pulse oximetry probe location. Mean, median, Q1 = 1st, Q2 = 3rd quartile of PWTT-measurements

**Fig. 4:**
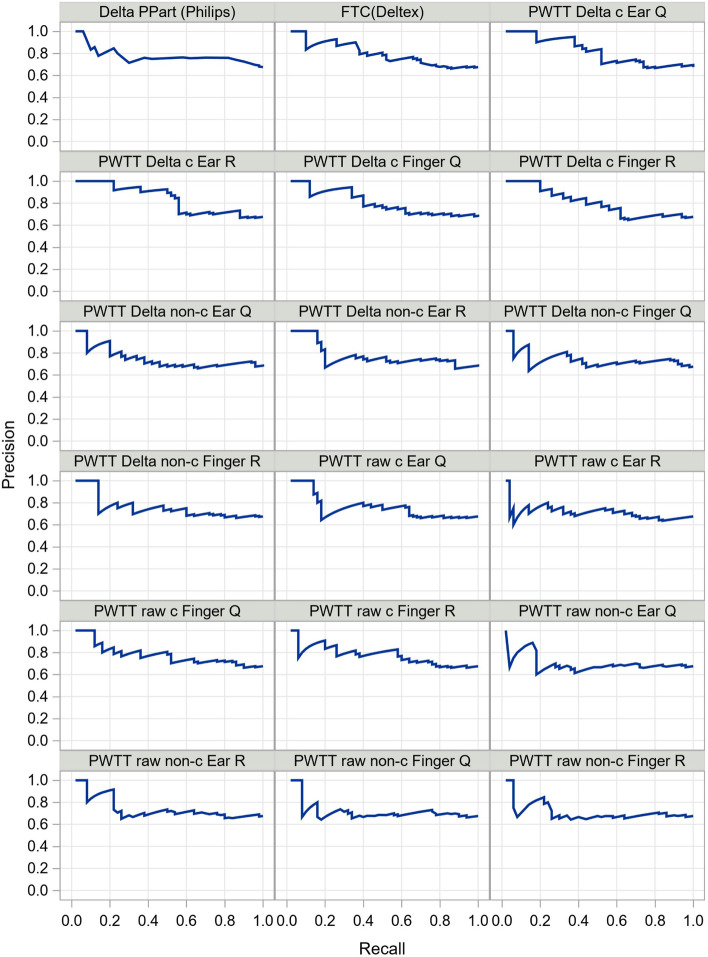
Precision-Recall curves of all 16 PWTT-parameters, ∆PP and FTc

Distribution of PWTT measurements before and after fluid bolus are illustrated by boxplots in Figs. 2 and 3. Mean, median, 1^st^ and 3^rd^ quartile of all measurements are shown in numbers for each PWTT-parameter underneath the corresponding boxplot.

### Secondary analysis: comparison of best-PWTT to ∆PP and FTc

Both ∆PP (AUC 0.65, SD 0.07, 95% CI 0.51–0.79) and FTc (AUC 0.62, SD 0.07, 95% CI 0.49–0.75) obtained results that were close to ‘best-PWTT’ for prediction of fluid responsiveness. Using the methods by DeLong [19] for comparing AUCs of correlated ROC curves, no significant difference for prediction of fluid responsiveness was observed for either method when compared with best-PWTT (*p* = 0.88 for ∆PP and *p* = 0.68 for FTc).

## Discussion

In this prospective clinical study we compared multiple ways of measuring and calculating the non-invasive monitoring parameter PWTT:

*Earlobe vs Fingertip* Even though several studies [9, 21] attached a pulse oximeter to the finger to obtain a photoplethysmographic wave for non-invasive flow-based assessment of fluid responsiveness, obtaining ear plethysmographic waveforms might be advantageous to monitor central hemodynamic changes, as measurements are less prone to errors due to vasoconstriction [22].

*Q- vs. R-Wave* Both, Q-Wave [10] as well as R-wave have been used in previous studies to define the start of the measurement period for non-invasive flow-based assessment of fluid responsiveness [9, 17, 21]. As the difference amount to only a few milliseconds, but identification of the R wave is more practical in most available monitors, this pragmatic approach could be widely implemented.

*Corrected vs. non-corrected* Not surprisingly, a correction to compare measurements at different heart rates improves the performance of a hemodynamic monitoring technology. The current clinical standard is the most widely used Bazett formula [16].

*Respiratory variation* A number of clinically established parameters to assess fluid responsiveness is based on heart–lung interaction [8]. So it has been shown for the parameters PEP [10] and PWTT [9]. However, other well-established flow-based hemodynamic monitoring technologies like esophageal doppler [12] do without an assessment of respiratory variation.

Consequently, we first identified one ‘best-PWTT’ approach achieving the highest AUC for prediction of fluid responsiveness, i.e. measuring PWTT by a pulse oximeter attached to the earlobe and using the R-wave in ECG, correcting by Bazett’s formula, and calculating the respiratory variation of PWTT (∆PWTT).

In the present study, the area under the ROC curve for ‘best-PWTT’ was 0.66 (SD 0.06, 95% CI 0.54–0.79). Accordingly, PWTT showed only poor ability as a diagnostic tool to discriminate fluid responders from non-responders intraoperatively. We hence were not able to confirm previous positive results from an ICU population [9] or more recent animal studies [23] in a clinical setting.

Second, after identifying ‘best-PWTT’ we compared the new flow-based monitoring parameter to established alternatives: The performance of ∆PP (AUC 0.65, SD 0.07, 95% CI 0.51–0.79) and FTc (AUC 0.62, SD 0.07, 95% CI 0.49–0.75) was not better when compared with best-PWTT.

Although several clinical studies in major abdominal surgery show an improved outcome of intraoperative fluid management monitored by esophageal doppler [24], the reported AUC of FTc to predict fluid responsiveness varies widely from an AUC of 0.49 (95% CI: 0.37–0.62) [25] to 0.94 (95% CI: 0.74–0.99) [26]. Two meta-analyses [13, 27] describe the AUC of ∆PP to predict fluid responsiveness to be 0.94 (95% CI 0.91 to 0.95) and 0.94 (95% CI 0.93 to 0.95), respectively. A lower AUC of 0.73 (95% CI 0.68–0.77) was reported in a pooled data-analysis of eight different ∆PP-studies in ICU patients receiving lung protective ventilation [28]. As in the present study patients were ventilated with a tidal volume ≥ 8 ml/kg IBW, the poor predictive performance of ∆PP for fluid responsiveness remains unclear.

This study has several limitations. First, all patients being studied had to have a stable sinus rhythm being mandatory for an assessment of PWTT. However, this applies also to ∆PP. Second, stroke volume measurements by esophageal doppler are affected by substantial variability between different observers [29]. To minimize this effect, esophageal doppler measurement was performed by one of the authors exclusively. Third, the effects of confounding factors on PWTT assessment, most probably height [30] but maybe also age or the presence of hypertension or peripheral arterial occlusive disease are largely unknown. Fourth, administering a fluid bolus of 7 ml/kg IBW may be considered a significant bolus. However, many studies evaluating fluid responsiveness use 500 ml of fluid as fluid challenge [31]. Finally, the most suitable site for pulseoximeter probe location (ear vs. fingertip) needs to be determined in larger studies. Due to the small numbers of patients, the study results can be considered as descriptive only.

## Conclusion

In conclusion, PWTT showed only poor ability to predict fluid responsiveness intraoperatively (AUC 0.66). Interestingly, established fluid responsiveness parameters ∆PP and FTc did not perform better in the present study population. Furthermore, F1-Scores suggest ∆PWTT to be a well-balanced parameter not leaning too much on sensitivity or positive predictive value alone. Being non-invasively measured in real time and without the need for cost-intensive additional monitoring devices PWTT comes along with well needed attributes in clinical routine. This is crucial, as even though at least one hemodynamic parameter to predict fluid responsiveness would be available in more than 90% of cases performing volume expansion, they are roughly used in a third [32]. Thus, clinical studies investigating this parameter using the presented ‘best-PWTT’ approach in a larger number of patients are desired. Analysing differences between low and high-tidal volume ventilation might reveal other benefits when compared to common fluid responsiveness parameters like ∆PP.

### Supplementary Information

**Additional file 1.** Best-PWTT dataset legend.

## Data Availability

All data generated or analysed during this study are included in this published article and its supplementary information files.

## Appendix

Figure 4

## Abbreviations

ASA-PS: American Society of Anesthesiologists Physical Status
AUC: Area under the ROC curve
BMI: Body mass index
BW: Body weight
CI: Confidence interval
CO: Cardiac output
ECG: Electrocardiography
EDM: Esophageal doppler monitoring
FTc: Corrected flow time
HF: Heart frequency
IBW: Ideal body weight
MAP: Mean arterial pressure
PEP: Pre-ejection period
PWTT: Pulse wave transit time
∆PWTT: Respiratory variation of PWTT
∆PP: Difference in pulse pressure
ROC-curve: Receiver operating characteristic curve
SD: Standard deviation
SV: Stroke volume
