# Supplementary material for: Non-invasive assessment of Pulse Wave Transit Time (PWTT) is a poor predictor for intraoperative fluid responsiveness: a prospective observational trial (best-PWTT study)
Source: BMC Anesthesiol. 2023 Feb 27;23:60. doi: 10.1186/s12871-023-02016-0 (PMC9969649; doi:10.1186/s12871-023-02016-0)
Supplement: Supplementary file 1 — Additional file 1. Best-PWTT dataset legend. [file 12871_2023_2016_MOESM1_ESM.zip › legend_dataset_bestPWTT.docx]

**Best-PWTT dataset legend**

raw measurements in [ms] vs. ∆= respiratory variation in [%] of PWTT

c = corrected measurements according to Bazett’s formula vs. non-c = uncorrected measurements

Q vs. R = start of PWTT-measurements with Q- or R-wave in ECG

finger vs. ear = pulse oximetry probe location

patient information = anonymous study code

HF = heart frequency [beats per minute]

MAP = mean arterial pressure [mmHg]

fTc = corrected flow time [ms]

SV = stroke volume [ml]

CO = cardiac output [l/min]

∆PP = pulse pressure variation [%] as measured by Philips IntelliVue MX700 or LifeScope® model J BSM-9101 Nihon Kohden

amount = amount of fluid bolus administered [ml]

SV- /CO- /fTc-Change = change of parameter 1 minute after each fluid bolus when compared to measurement before fluid bolus [%]
